# *Fusarium*: Molecular Diversity and Intrinsic Drug Resistance

**DOI:** 10.1371/journal.ppat.1005464

**Published:** 2016-04-07

**Authors:** Abdullah M. S. Al-Hatmi, Jacques F. Meis, G. Sybren de Hoog

**Affiliations:** 1 CBS-KNAW Fungal Biodiversity Centre, Utrecht, the Netherlands; 2 Institute of Biodiversity and Ecosystem Dynamics, University of Amsterdam, Amsterdam, the Netherlands; 3 Directorate General of Health Services, Ministry of Health, Ibri Hospital, Ibri, Oman; 4 Department of Medical Microbiology and Infectious Diseases, Canisius Wilhelmina Hospital, Nijmegen, the Netherlands; 5 Department of Medical Microbiology, Radboud University Medical Center, Nijmegen, the Netherlands; 6 Basic Pathology Department, Federal University of Paraná State, Curitiba, Paraná, Brazil; 7 Biological Sciences Department, Faculty of Science, King Abdulaziz University, Jeddah, Saudi Arabia; Duke University Medical Center, UNITED STATES

## The Increasing Incidence of Fusariosis

The fungal genus *Fusarium* contains an extraordinary genetic diversity and is globally distributed in plants, soil, water, and manmade habitats. Plant-pathogenic members of the genus cause diseases in many agriculturally important crops, with billions of dollars of economical losses annually. Their presence as food contaminants is detrimental because of production of biologically active, highly toxic secondary metabolites.

Remarkably, human fusariosis does not have a long history. This is in contrast to comparable opportunistic pathogens like *Scedosporium* spp., which have been known since the 19th century. The first case of human *Fusarium* infection was reported only in 1958 and concerned an eye infection caused by a blow from a cow tail [1]. *Fusarium* causes a very wide spectrum of diseases, ranging from mildly superficial to fatally disseminated [2]. During the initial years, most reported infections were caused by traumatic inoculation. Keratitis is still the most common infection by *Fusarium* species, occurring especially in the warmer climates of India, China, and Brazil [3]. After 1960, the increasing use of antibiotics became a major predisposing condition [4]. Since 1970, prolonged neutropenia due to intensified cytotoxic treatment of hematologic malignancies was the leading risk factor in novel types of fusariosis [5]. Since 1980, *Fusarium* infections have been seen in severely immunocompromised patients with a 100% mortality rate, e.g., in cases of cerebral involvement [6]. Today, invasive surgery, organ transplantation, chronic steroid treatment, and aggressive cytotoxic therapy are the main risk factors of fusariosis [7]. *Fusarium* shows a dynamic response to opportunities provided by underlying disorders of the host.

## Long-Distance Dispersal of Opportunists

Emergence of infectious diseases in humans might be expected to be caused by host shifts from animal reservoirs to humans [8], as is the case, e.g., in dermatophytes. *Fusarium* is different. Plant pathogens are dispersed via direct contact, wind, water, vectors such as insects, or the germline of contaminated seeds [9]. Host ranges can be broad or narrow; some species seem to be host-specific (e.g., *Fusarium ficicrescens* on figs), while others are found on widely different hosts (e.g., *Fusarium oxysporum*). Some species are associated with specific geographic areas (e.g., *Fusarium lactis* in California), but most fusaria are ubiquitous [10]. It is possible that outbreaks leading to repeated isolation from the same host plant masquerades as host-specificity; for most species, plant inoculation experiments have not been done and ecological specialization has not been proven.

**Fig 1:**
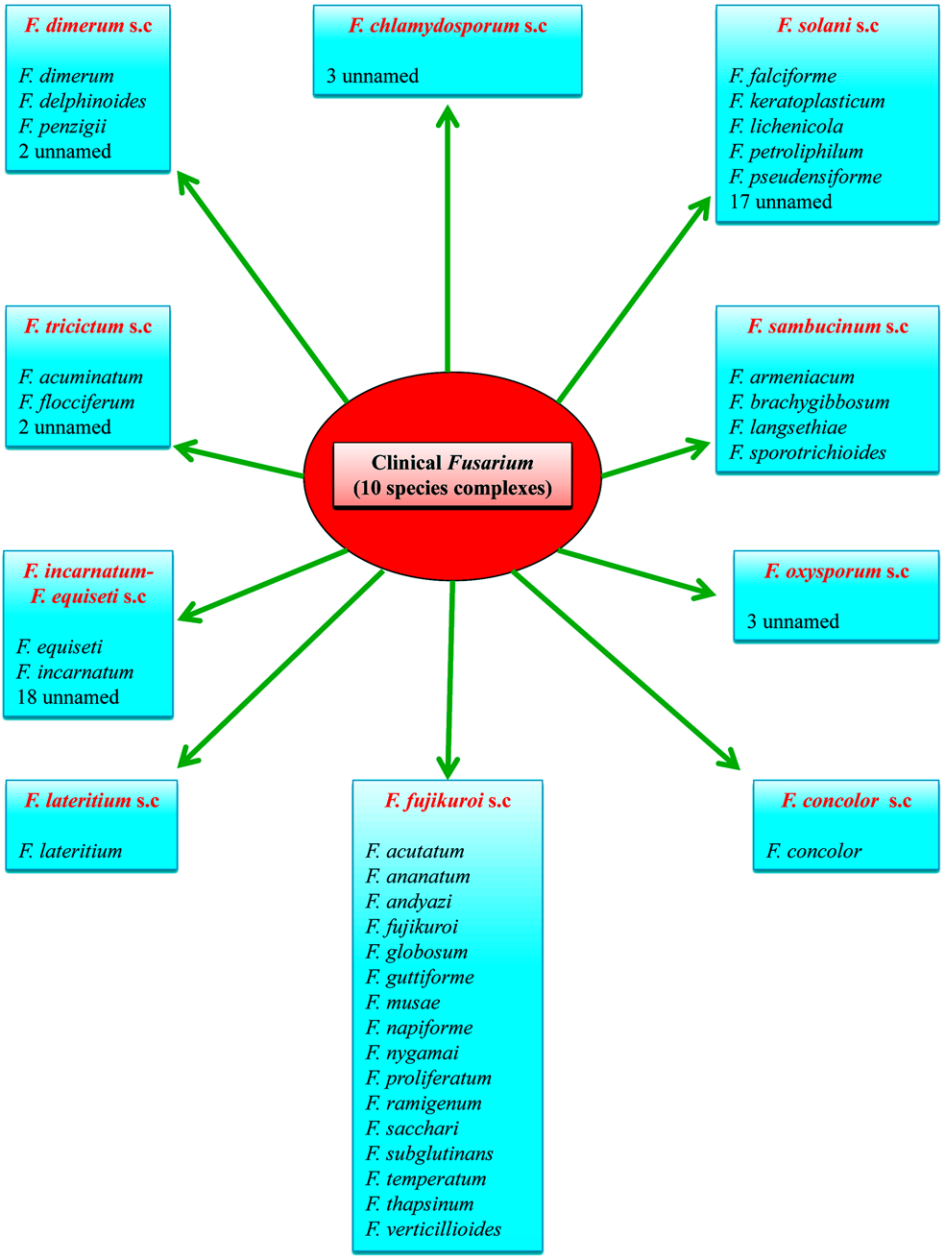
Schematic representation illustrating up-to-date clinical *Fusarium* species reported from clinical cases and belonging to ten *Fusarium* species complexes.

The genus *Fusarium* was first described in the early 19th century. In 1935, Wollenweber and Reinking used morphological differences to organize the genus into 16 sections with 65 species, 55 varieties, and 22 forms [11], but later Booth simplified this to only 14 species [12]. When Leslie and Summerell used morphological and phylogenetic information, they ended up with 70 species, most of which formed falcate, multiseptate macroconidia with a beaked apex and a pedicellate basal cell. The microconidia are one- to two-celled and pyriform, fusiform, or ovoid in shape. Both macro and microconidia are produced in the aerial mycelium on phialides [13]. At present, with the dawn of molecular sequencing, more than 200 species are recognized in 22 species complexes, differing by morphology, host association, and molecular parameters [14]. Currently, 74 taxonomic species have been suggested to cause human infections (Fig 1) [15], judging from their isolation from clinical samples, and this number is expanding. To date, about 36 of the alleged human opportunists carry a name, while 38 are still unnamed and can only be identified by multilocus sequence analysis (MLSA). Thus far, 21 species have been described with proven case reports [16], and more have been published in the literature.

The most common route of human infection is by inoculation via contaminated thorns or plant leaves, which particularly affects farmers and agricultural workers. However, *Fusarium* poses a challenge for human disease management because propagules may disperse over long distances in the atmosphere, and new resources and susceptible hosts are quickly found [17]. Smith et al. [18] noted that *Fusarium* conidia are waterborne and become airborne when dried. Schmale et al. [19] showed that large-scale atmospheric features known as Lagrangian coherent structures (LCSs) enhance transport of *Fusarium* in the lower atmosphere. Lin et al. [20] demonstrated that atmospheric populations of fusaria are mixed and that conidial counts do not vary across consecutive sampling intervals, demonstrating constant airborne transport.

One of the major risk factors for compromised hosts is inhalation of contaminated air. Moretti et al. [21] established a link between *Fusarium* in the air and in the blood of infected patients, and suggested unfiltered hospital air may be problematic for these patients. Short et al. [22] investigated hospital plumbing systems for the occurrence of fungi and found that these systems are a hidden reservoir for *Fusarium*. Prevalence of *Fusarium* species in compromised patient populations is not proportional to their environmental abundance [23], suggesting that infection is not merely a random process.

## Prevalence of *Fusarium* Antifungal Resistance

*Fusarium* species are intrinsically resistant to azole antifungals. Five azole fungicides are widely used for plant protection: propiconazole, bromuconazole, epoxiconazole, difenoconazole, and tebuconazole. Azoles are generally inexpensive and have broad-spectrum activity and long stability. Azoles that are used clinically have derivatives such as imidazole or triazole rings [24]. The azoles used in agriculture are different, but all azoles target the same active site, i.e., lanosterol-14α-demethylase [25]. Effects on *Fusarium* population dynamics in agricultural fields are likely due to decreased competition with susceptible species [26]. This may be reinforced by antifungal prophylaxis in high-risk patients in clinical settings, enhancing selective pressure that favours multidrug-resistant fungi, including *Fusarium* [27]. Population dynamic effects have not been seen before because *Fusarium* was not considered to be a matter of concern [28]. The growing incidence of severe human *Fusarium* infections may change this situation.

## Multiresistance Against Different Classes of Antifungals

Clinically relevant members of *Fusarium* are resistant to almost all currently used antifungals—not only azoles, but also echinocandins and polyenes. This poses a major challenge to medicine and agriculture, particularly with emerging and globally spreading fungi like *Fusarium*. There are only a few options left for treating patients and crops. Intrinsic, primary resistance is found naturally among some *Fusarium* species without prior exposure to the drug [29]. Secondary resistance to azoles develops among previously susceptible strains after exposure to the antifungal agent, as seen, e.g., in *Aspergillus fumigatus*, and is usually dependent on altered expression of CYP51, the gene encoding sterol 14α-demethylase [30]. Recently, Fan et al. showed that CYP51 in *Fusarium* has three paralogues (CYP51A, -B, and -C), with CYP51C being unique to the genus [31]. CYP51A deletion usually causes secondary resistance in fungi such as *A*. *fumigatus* [25], whereas the opposite was found in *Fusarium*: CYP51A deletion increases the sensitivity of *Fusarium graminearum* to azoles and other fungicides (prochloraz, tebuconazole, and epoxiconazole) that are used in plant protection [31]. The exact resistance mechanisms in *Fusarium* are not entirely understood, but combinations of CYP51A amino acid alterations and/or CYP51A gene overexpression might be involved.

**Fig 2:**
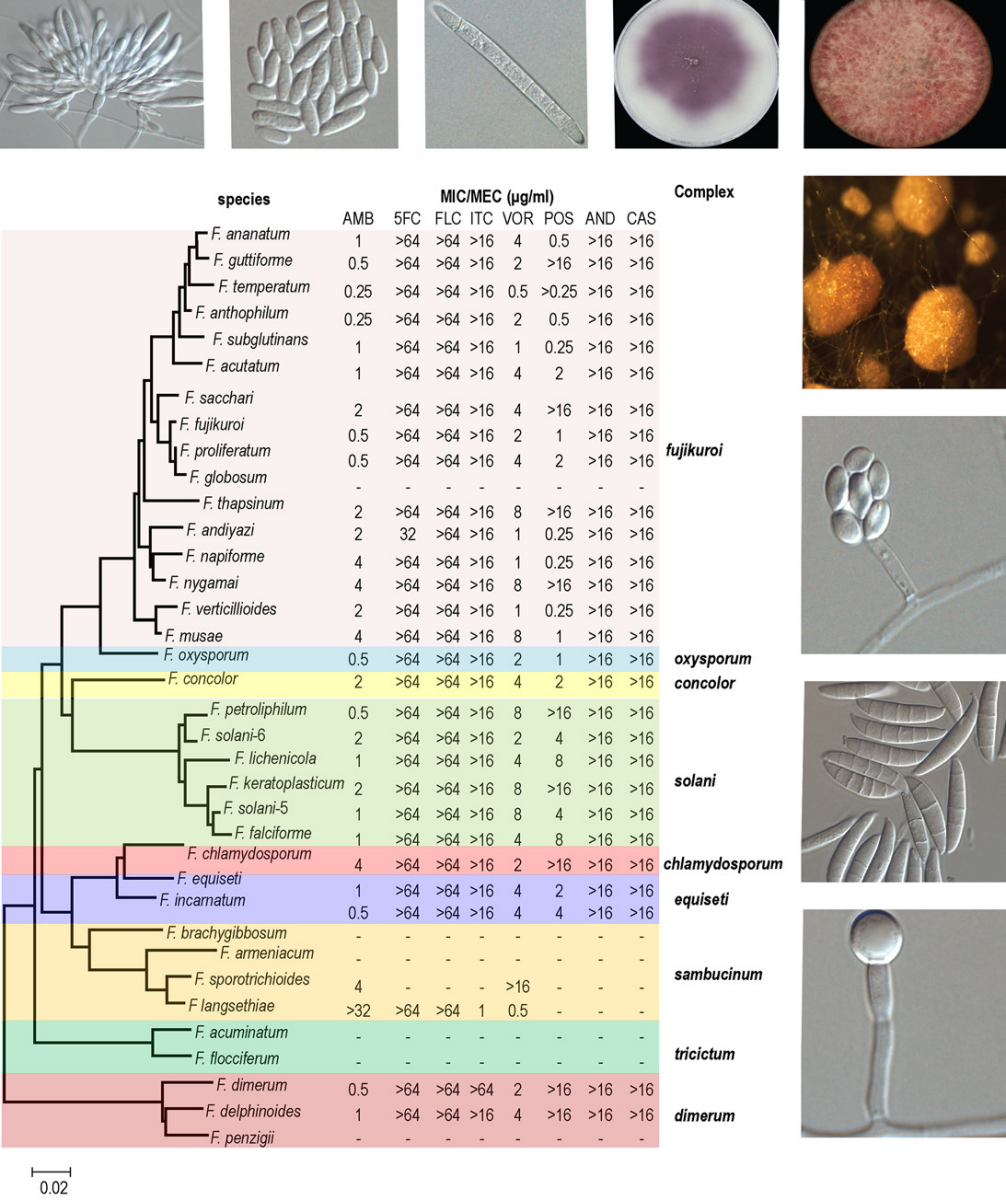
Sequence-based phylogeny of clinically related *Fusarium* species and associated antifungal susceptibilities with morphological features. Neighbor-Joining tree created by MEGA6 from *TEF1* sequences of clinically related *Fusarium* species using 1,000 bootstrap replicates. The minimum inhibitory concentration (MIC) profiles of eight antifungals against each species have been incorporated into the figure. “-,” no data available for these species. AMB = amphotericin B, FLC = fluconazole, ITC = itraconazole, VOR = voriconazole, POS = posaconazole, CAS = caspofungin, 5FC = 5-flucytosine, AND = anidulafungin.

Recent findings indicate that a mutation occurring in the FKS1 gene might contribute to the intrinsic echinocandin resistance in *Fusarium*. In support of this, evidence has been presented that hot spot 1 substitution P647A and F639Y in FKS1 contribute to resistance of *Fusarium solani* [32]. Furthermore, *Fusarium* has an effective efflux mechanism to remove xenobiotics from its cells [33], and this may also reduce azole sensitivity. Amphotericin B, second-generation broad spectrum triazoles (fluconazole, itraconazole, voriconazole, and posaconazole), antimetabolites (5-fluorocytosine), and echinocandins (caspofungin, anidulafungin, and micafungin) all have limited activity against *Fusarium* species. High-level cross-resistance to fluconazole and itraconazole was reported in almost all *Fusarium* species. Cross-resistance has been observed among the three echinocandins in *Fusarium* species (Fig 2). In vitro data showed that there is potential for azole cross-resistance to echinocandins and polyenes, but no clinical information on this phenomenon is available.

## Management of *Fusarium* Infections: New Drugs or Drug Combinations?

Treatment of fusariosis is a major challenge. For disseminated fusariosis in immunocompromised patients, the 12-week survival time has increased significantly in the last decade in single center studies [34], national studies, [35] and worldwide evaluations [36]. This better outcome of treatment is probably associated with the introduction of voriconazole in 2002. Therapy with amphotericin B deoxycholate gives poor survival results of 28% when compared to lipid amphotericin B (53%) or voriconazole (60%) [36]. Recent European guidelines [37] suggest treating disseminated fusariosis with voriconazole and lipid amphotericin B, although evidence is based on expert opinion and case series rather than on clinical trials. Of the azoles, only the new triazoles, voriconazole and posaconazole, show moderate activity, with mode minimal inhibitory concentrations (MICs) of 2–8 mg/L and 0.5–8 mg/L, respectively, depending on the species complex. The mode MIC of amphotericin B is 2 mg/L irrespective of the species complex (Fig 2) [38]. The newest class of antifungal drugs, the echinocandins, have activity against *Candida* and *Aspergillus* species [30], but for *Fusarium* they appear to be inactive with high MICs of >16 mg/L (Fig 2). Terbinafine is another option to treat some *Fusarium* species, but this compound is only registered to treatment of superficial infections [39]. Natamycin (5%) and/or topical amphotericin B (0.5%) are first-line treatment of fungal keratitis in some countries. Elsewhere, topical 1% voriconazole and/or 5% natamycin are used for this type of infection [40].

Considering the poor outcome obtained with monotherapy, attempts have been made to determine whether combinations of drugs lead to improved efficacy. Spader et al. [41] reported that synergistic interactions were observed for the combinations of amphotericin B with caspofungin (68.7%), amphotericin B with rifampin (68.7%), amphotericin B with 5-flucytosine (59.3%), and amphotericin B with voriconazole (37.5%). Al-Hatmi et al. [42] determined in vitro antifungal activity of natamycin alone and in combination with voriconazole for *Fusarium* keratitis, and found that MICs of these compounds alone were >4 and 4−8 mg/L, respectively, and that the combinations tested displayed (70%) in vitro synergistic effects against a significant number of isolates. MICs values were reduced to 0.02−0.5 mg/L and to 0.13−2 mg/L in combination, respectively [42]. Combinations of voriconazole, amphotericin B, and posaconazole showed poor efficacy in experimental murine infections by *F*. *verticillioides*, while the combination of liposomal amphotericin B and terbinafine showed good results [23]. However, clinical studies have not been performed, and the most efficacious combination remains to be explored. Further work on interactions in animal models or clinical trials with the aim to obtain higher cure rates of *Fusarium* infections is overdue.
